# Recent developments in tau-based therapeutics for Alzheimer’s disease and related dementsia

**DOI:** 10.1186/2193-1801-4-S1-L14

**Published:** 2015-06-12

**Authors:** Miguel Medina

**Affiliations:** CIBERNED and CIEN Foundation, Madrid, Spain

**Keywords:** Tau, Alzheimer, dementia

Current therapies for Alzheimer’s disease (AD) and related disorders have demonstrated very modest, symptomatic efficacy, leaving an unmet medical need for new, more effective therapies. While drug development efforts in the last two decades have primarily focused on the amyloid cascade hypothesis, with disappointing results so far, tau-based strategies have received little attention until recently despite that the presence of extensive tau pathology is central to the disease. The discovery at the turn of the century of mutations within the tau gene that cause fronto-temporal dementia demonstrated that tau dysfunction was per se sufficient to cause neuronal loss and clinical dementia. Development of tau pathology is associated with progressive neuronal loss and cognitive decline and is the common underlying cause of a group of neurodegenerative disorders collectively known as “tauopathies”. Tauopathies are clinically, morphologically and biochemically heterogeneous neurodegenerative diseases characterized by the deposition of abnormal tau protein in the brain. The neuropathological phenotypes are distinguished based on the involvement of different anatomical areas, cell types and presence of distinct isoforms of tau in the pathological deposits. Thus, the spectrum of tauopathy entities expands beyond the traditionally discussed disease forms. Emerging evidence strongly suggests that accumulation of abnormal tau is mediated through spreading of seeds of the protein from cell to cell. This prion-like mechanism would support the concept that in AD brains, tau pathology iinitiates in a very small part of the brain many years before becoming symptomatic, spreading slowly and progressively to the whole brain following an anatomically defined pattern. Emerging therapeutic strategies aimed at treating the underlying causes of the tau pathology will be discussed, including some novel therapeutic approaches on the verge of providing new treatment paradigms in upcoming years.

